# The Minister of Pensions and Poor-Law Infirmary Accommodation

**Published:** 1918-08-17

**Authors:** G. W. Tarratt

**Affiliations:** Secretary. County Borough of Walsall, Naval and Military War Pensions, etc., Local Committee. Temporary Offices : The Bridge, Walsall.


### The Minister of Pensions and Poor-Law Infirmary Accommodation.

### THE CASE OF ERNEST SEVIER.

We have received the following letter, which relates to the first news paragraph on page 359 of The Hospital of July 27:—

The Secretary, Ministry of Pensions,
Westminster House, Millbank,
London, S.W. 1.

*Re* Ernest Sevier, Pte., 40600, 3rd Suffolk Regt.

Sir,—With further reference to the above mental case, and your inquiry of the 29th ulto., I am requested by my Health Committee to forward to you the full particulars, viz.: On July 9 last I received letter dated July 1 from this man's mother, sent to the Commanding Officer, stating that she could not undertake to receive her son at home, as she had no one to look after him. I also received letters from Lt.-Col. Frankey (C.O.), dated the 4th and 5th inst., stating that this man's discharge had been approved as a "harmless lunatic," and asking that the man be received into an institution of the borough. A further letter was received, dated July 12, asking for the decision

My committee considered, in view of the very difficult nature of the case, to instruct the C.O. that the man be sent to the Walsall Infirmary for observation, as we had no other available institution where the man could be sent to for this purpose. On July 13 I wrote to the C.O. in the following terms: "The matter has been before my committee, who, in the man's own interests, have decided that he should be first admitted to the Walsall Infirmary, instead of straight to Burntwood Asylum, for the present, and it is hoped accommodation may afterwards be found for him in a suitable institution." He arrived under escort on July 22, and after being at the infirmary for a very short time was found to be worse than was anticipated, and accordingly was then duly certified and removed to Burntwood Asylum.

My committee are fully alive to the interests of the discharged men, and quite recently decided, in view of Circular 79 to issue instructions that discharged men were not to be taken to the infirmary, only in very exceptional cases, and then only for a short time for observation purposes, to avoid, if possible, any asylum taint being attached to the man. They claim that the arrangements made were in the best interests of the man.

I am instructed to forward a copy of this letter to The Hospital.—Yours faithfully, G. W. Tarratt,
County Borough of Walsall, Secretary.
Naval and Military War Pensions, etc.,
Local Committee.
Temporary Offices: The Bridge, Walsall.
August 3, 1918.

[The above letter demonstrates the urgency which exists for pressure by Mr. Hodge, the Minister of Pensions, upon Naval and Military War Pensions Local Committees, and especially on that of the County Borough of Walsall. This is shown by the wording of the last paragraph but one, which demonstrates that the Walsall Local Committee had issued instructions that discharged men may be taken to the Poor-Law infirmary in exceptional cases for a short time. The preceding paragraph demonstrates the ill-effects to the warrior and, apart from the pauper taint, the wrongness of sending to a Poor-Law infirmary any such cases as that of Ernest Sevier, late private in the Suffolk Regiment, for this poor man, after being insulted by the course taken, had to be duly certified and removed to an asylum. Sevier's case ought surely to make it impossible for the Minister of Pensions longer to permit Local Committees to use Poor-Law establishments at any time in any way for the reception of warriors discharged from service but requiring medical or other care and accommodation. We once more call Mr. Hodge's attention to our note on p. 359 of The Hospital of July 27, and shall hope to hear that he has applied the necessary remedy by restraining every Local Committee from again following a course similar to or identical with that pursued by Walsall in the case of Ernest Sevier.—Ed. The Hospital.]

